# Effects of a Monophasic Hormonal Contraceptive With Norgestimate+Ethinyl Estradiol on Menstrual Bleeding: Protocol and Design of a Multicenter, Prospective, Open-Label, Noncomparative Study in Italy

**DOI:** 10.2196/63683

**Published:** 2025-03-31

**Authors:** Angelo Cagnacci, Giovanni Grandi, Giampiero Capobianco, Anna Maria Fulghesu, Giuseppe Morgante, Vincenzo Biondelli, Elena Piccolo, Elena Casolati, Mario Mangrella

**Affiliations:** 1 Dipartimento di Neuroscienze, Riabilitazione, Oftalmologia e Scienze Materno Infantili Istituto di Recerca e di Cura e Carattere Scientifico (IRCSS)-Ospedale San Martino Genoa Italy; 2 Dipartimento di Scienze Mediche e Chirurgiche Materno-Infantili e dell'Adulto Azienda Ospedaliero—Universitaria di Modena Modena Italy; 3 Dipartimento di Scienze Mediche, Chirurgiche e Sperimentali Università degli Studi—Azienda Ospedaliero Universitaria di Sassari Sassari Italy; 4 Dipartimento di Scienze Chirurgiche Policlinico Universitario Monserrato Duilio Casula Monserrato Italy; 5 Dipartimento della Mamma e dei Bambini Unità Operativa Semplice Procreazione Medicalmente Assistita del Policlinico Le Scotte Siena Italy; 6 Unità Operativa Ostetricia e Ginecologia Ospedale San Pio da Pietrelcina Vasto Italy; 7 Medical Affairs Department Italfarmaco SpA Milan Italy; 8 Private Practice of Obstetrics and Gynecology Milan Italy

**Keywords:** combined oral contraceptive, ethinyl estradiol, menstrual cycle, monophasic, norgestimate, hormonal contraceptive, menstrual health, Italy, women’s health, patient-reported outcomes, methodology, observational study, reproductive health, data analysis, assessment

## Abstract

**Background:**

Norgestimate (NGM) is a progestin with negligible androgenic activity that is available in combination with ethinyl estradiol (EE) as a monophasic combined oral contraceptive (COC). It has been more than 30 years since a clinical study evaluated the effects of monophasic NGM/EE on menstrual cycle characteristics in healthy women, and in the interim, there has been growing recognition that clinical trials of contraceptives should evaluate a wide range of potential positive and negative impacts for users.

**Objective:**

The aim of this study is to investigate menstrual cycle control during the use of a monophasic COC formulation containing NGM 0.25 mg and EE 0.035 mg (Effimia; Italfarmaco SpA), using established methodologies as well as patient-reported outcomes.

**Methods:**

This is a prospective observational study being undertaken in a target population of 228 healthy Italian women aged 18-35 years who are starting oral contraception for the first time or switching from another COC. The participants are asked to complete a diary for 6 cycles recording information about their menstrual cycles (frequency, duration, regularity, estimated flow volume, and breakthrough bleeding), any unscheduled bleeding, and an evaluation of dysmenorrhea, using a 100-mm visual analog scale from 0=no pain to 100=very severe pain, and any adverse events. Compliance is assessed after 3 and 6 months via returned medication. The primary end point is the change from baseline in the rate of intermenstrual bleeding during the sixth cycle. At baseline, 3 months, and 6 months, acne will also be assessed using the Global Acne Grading Scale, and participants will complete a Profile of Mood State to assess premenstrual syndrome and the Female Sexual Function Index to evaluate the quality of their sex life. A subgroup of 28 participants at 1 site (Genoa) is also providing a blood sample for the assessment of metabolic, endocrine, and coagulation parameters.

**Results:**

Study enrollment began in July 2023 and is expected to be complete by December 2024. Data analysis is expected to be complete by October 2025.

**Conclusions:**

This study into the effects of monophasic NGM/EE 0.25/0.035 mg on menstrual characteristics in healthy Italian women will provide up-to-date data on these effects and includes assessments of a range of other parameters, such as acne severity and patient-reported outcomes, in line with recent international consensus recommendations.

**Trial Registration:**

ClinicalTrials.gov NCT06067256; https://clinicaltrials.gov/study/NCT06067256 and EudraCT 2021-003027-15; https://www.clinicaltrialsregister.eu/ctr-search/trial/2021-003027-15/IT

**International Registered Report Identifier (IRRID):**

DERR1-10.2196/63683

## Introduction

Combined oral contraceptives (COCs) are the most commonly prescribed form of hormonal contraception in Italy and in other European countries [1]. These contraceptive pills contain a combination of estrogen and progestin to suppress ovulation (the primary contraceptive effect) and to change endometrial and cervical secretions, hindering the passage of sperm [2]. COCs may be monophasic or multiphasic. Monophasic COCs contain the same dose of estrogen and progestin in each pill, whereas the ratio of these hormones varies over the course of a cycle in multiphasic COCs.

Since the first development of COCs in the 1960s, formulations have been modified to reduce the risk of undesirable adverse events (AEs) and cardiovascular health risks [3]. The early progestins used in first- and second-generation COCs were chemically related to testosterone and caused androgenic AEs such as acne, hirsutism, and oily skin, which could undermine adherence as well as negative effects on high-density lipoprotein cholesterol [4]. Newer progestins have been structurally modified to be less androgenic or are structurally related to spironolactone [5,6]. In addition, estrogen doses have been progressively lowered, and most COCs in use today contain ≤0.035 mg of ethinyl estradiol (EE) [3]. COCs containing the first- and second-generation progestins were associated with a markedly higher risk of venous thromboembolism (VTE) compared with COCs containing the newer-generation progestins [7].

One such newer-generation progestin is norgestimate (NGM). NGM is a third-generation progestin derived from 19-nortestosterone [8]. It is rapidly hydrolyzed in vivo to norelgestromin, the primary active metabolite (75% to 80%), and levonorgestrel (20% to 25%) [9]. Both NGM and its metabolites have activity similar to endogenous progesterone but have a very poor affinity for androgen receptors and are therefore less androgenic than earlier progestins such as norgestrel, gestodene, or levonorgestrel [9,10]. In addition, unlike levonorgestrel, NGM increases levels of sex hormone–binding globulin in plasma, but does not bind to sex hormone–binding globulin, and is associated with reduced levels of free testosterone [11]. Data show that COCs containing NGM carry a lower risk of VTE than COCs containing cyproterone acetate, desogestrel, dienogest, drospirenone, or gestodene [12,13]. On the other hand, contraceptive patches containing norelgestromin are associated with a slightly higher incidence of VTE compared with levonorgestrel-containing COCs, presumably because of the high cumulative dose of EE that accompanies the transdermal route of administration [9].

Women want their contraceptives to be effective in preventing pregnancy, convenient, safe, and well tolerated, and with excellent cycle control [14]. Unscheduled bleeding has a number of negative impacts on a woman’s life, in addition to the inconvenience, including the potential for a negative effect on their sexual life and confusion about the status of their cycle [14]. Some women may interpret unscheduled bleeding as an indication that the COC is not working or interrupt use because of it [14]. Monophasic COCs containing 0.030-0.035 mg of EE offer women the greatest likelihood of a regular bleeding pattern with a low risk of estrogen-associated risks as well as the option of fewer periods by reducing or eliminating the placebo period altogether [15]. As well as preventing pregnancy and potentially providing better cycle control (increased regularity and reduced intermenstrual bleeding), COCs may also offer improvements in acne and premenstrual or menstrual symptoms, such as mood changes, headaches, and pelvic pain [16]. However, effects on these parameters vary between COCs [16].

A large German study (N=59,701) demonstrated that the use of a monophasic COC containing 0.25 mg of NGM and 0.035 mg of EE reduced the rate of breakthrough bleeding in healthy women compared with the rate before they started taking NGM/EE [17]. Similar results were seen in an Italian study among 92 women receiving NGM/EE [18]. However, both of these trials were conducted more than 30 years ago, and there has been no recent research in Italy to confirm the effects of monophasic NGM/EE in healthy women. Moreover, since then, there has been growing recognition that clinical trials of contraceptives should evaluate a wide range of potential positive and negative impacts for users [19].

Thus, this study is being undertaken to investigate menstrual cycle control during the use of a monophasic COC formulation containing NGM/EE 0.25/0.035 mg (Effimia; Italfarmaco SpA), using established methodologies as well as patient-reported outcomes (PROs).

## Methods

### Recruitment

This prospective, open-label study is being conducted at 6 centers in Italy in healthy women aged 18 to 35 years who are starting oral contraception for the first time or planning to switch to a new COC for the purpose of contraception and not for therapeutic reasons. No specific tests are performed before enrollment, consistent with Italian guidelines that such tests are not indicated in women without individual or family risk factors [20]. Patients who are switching need to undergo a 1-month washout phase prior to entry by discontinuing their previous COC 1 month prior to starting NGM/EE 0.25/0.035 mg. The participants must be residents of Italy and be sufficiently proficient in Italian to understand the informed consent form and the instructions for COC use. The key exclusion criteria are contraindications to the COC according to the current summary of product characteristics, concomitant conditions that place users at risk of AEs, or the use of COC for indications other than contraception, such as polycystic ovarian syndrome, endometriosis, or recurrent menometrorrhagia. Complete inclusion and exclusion criteria are shown in Textbox 1. Certain concomitant medications are not permitted for the duration of the study to minimize the potential for drug interactions or confounding of end-point assessment (Textbox 1).

**Complete inclusion and exclusion criteria.**

**Inclusion criteria (all must be met)**

- Healthy women aged between 18 and 35 years (inclusive) in need of contraception.
- Residing in Italy and having a good knowledge of the Italian language, such as to correctly understand the informed consent form and the instructions for use and to ensure potential adherence to the study.
- Willing and able to understand and complete the written informed consent form.
- Willing to comply with the study protocol.

**Exclusion criteria (any 1 of these disqualifies an individual from participating)**

- Any contraindications to the use of combined oral contraceptives (COCs) according to the current summary of product characteristics of Effimia, that is, women presenting (or having ever presented) with myocardial infarction, transient ischemic attack, stroke, angina pectoris, deep vein thrombosis, pulmonary embolism (or presence of blood clots in organs other than legs and lungs), any blood clotting disorder (such as protein C deficiency, protein S deficiency, and antithrombin-III deficiency), or who need to undergo surgery or lie down for a long period of time (including the risk of previous deep vein thrombosis, arterial thromboembolism, hypertension in the course of treatment, and diabetes). If any of the listed conditions should appear during the use of the tested COC, the product must be stopped immediately, and the participant withdrawn from the study.
- Severe diabetes with blood vessel damage, heart valve disease with complications, severe hypertension, severe hypercholesterolemia or hypertriglyceridemia, hyperhomocysteinemia, migraine with aura, hepatitis C (and taking medications for this condition), endometrial hyperplasia, or unexplained vaginal bleeding.
- Women who are breastfeeding or pregnant or suspect they are pregnant.
- Current or history of any liver disease not yet recovered (liver function not yet normalized), any benign or malignant tumor of the liver, any breast or genital organs cancer (even suspected), or jaundice during pregnancy or while using hormonal contraceptives.
- Galactose intolerance, total lactase deficiency, or glucose-galactose malabsorption syndrome.
- Hypersensitivity to the active substances or to any excipients of the tested COC (eg, norgestimate, ethinyl estradiol, or lactose).
- Use of any of the following during the study period (according to the summary of product characteristics of Effimia): treatments for tuberculosis (eg, rifampicin), epilepsy (eg, primidone, phenytoin, barbiturates, carbamazepine, or oxcarbazepine), HIV and hepatitis C virus infection (protease inhibitor drugs and nonnucleoside reverse transcriptase inhibitors such as ritonavir, nevirapine, efavirenz, and also ombitasvir, paritaprevir, or dasabuvir), fungal infections (eg, griseofulvin), arthritis, osteoarthritis (etoricoxib), or pulmonary arterial hypertension (bosentan), and St John's wort used as an antidepressant. Medicines containing cyclosporine, antiepileptic lamotrigine, tranexamic acid, theophylline (used to treat respiratory problems), and tizanidine (used to treat muscle pain or cramps) should not be taken as well.
- Use of hormonal contraceptives in the previous month.
- BMI ≥30 kg/m^2^ (class I obesity).
- Smoking >15 cigarettes per day.
- Off-label use of COC (eg, for polycystic ovarian syndrome, endometriosis, or recurrent menometrorrhagia).
- Currently taking part or who took part in clinical studies with experimental products in the previous month.
- Incapacity or inability to comply with the study protocol (unreliability in the intake of the product or in the completion of the diary) according to the investigator’s opinion.

### Ethics Approval

The study protocol and amendments were approved by the ethics committee at the coordinating center (Territorial Ethics Committee—Liguria; application 202100302715-003), with the latest version approved on June 17, 2024. The study is being conducted in accordance with the Declaration of Helsinki (seventh revision, 2013), the Convention of Oviedo in April 1997 and the additional protocol in January 1998, and the national laws, regulations, and applicable guidelines of Italy. In addition, the study is consistent with the International Conference on Harmonization Tripartite Guidelines for Good Clinical Practice requirements. Prior to any procedures, participants are given information about the study and asked to sign an informed consent form. Their personal data are deidentified before being stored and protected according to the current European General Data Protection Regulations. The women do not receive any financial compensation for participating in the study.

### Procedures

**Figure 1:**
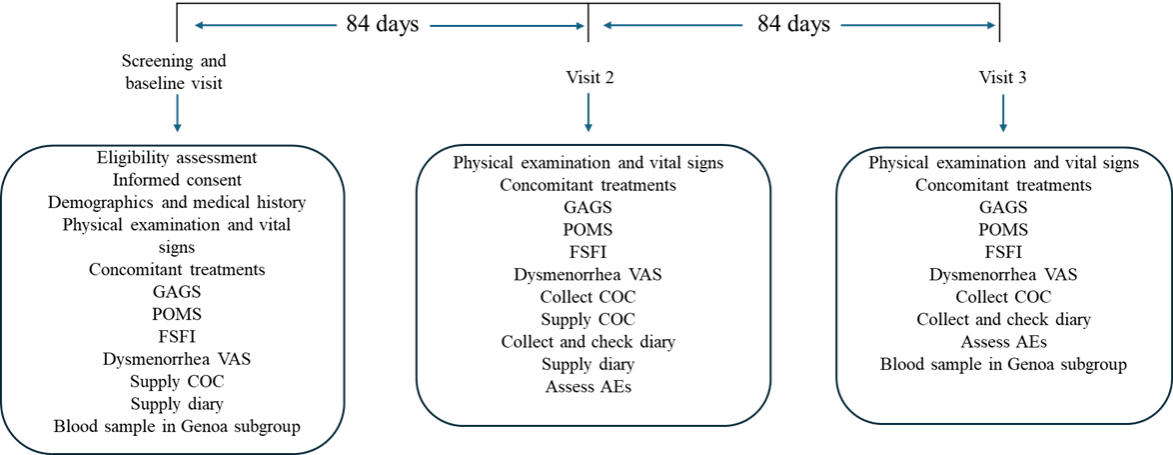
Study design. AE: adverse event; COC: combined oral contraceptive; FSFI: Female Sexual Function Index; GAGS: Global Acne Grading Scale; POMS: Profile of Mood State; VAS: visual analog scale.

During the baseline visit, participants have a physical examination, including an assessment of acne severity (using the Global Acne Grading Scale [GAGS] [21]), and provide a complete medical and menstrual history (Figure 1). They are asked about the characteristics of their menstrual cycle (frequency, duration, regularity, estimated flow volume, and breakthrough bleeding), any unscheduled bleeding, and an evaluation of dysmenorrhea, using a 100-mm visual analog scale (VAS) from 0=no pain to 100=very severe pain. Participants are also being asked to complete a Profile of Mood State (POMS) to assess premenstrual syndrome and the Female Sexual Function Index (FSFI) to evaluate the quality of their sex life, using the validated Italian language versions of these questionnaires [22-24]. At 1 site (Genoa), 28 participants are also providing a blood sample for the assessment of metabolic, endocrine, and coagulation parameters (Textbox 2).

At the baseline visit, participants are provided with NGM/EE 0.25/0.035 mg for the first 3 cycles and a diary, in which they are asked to record cycle evaluation parameters (Table 1), their medication-taking behavior, concomitant medications including over-the-counter medications (start and stop dates and dose), and any AEs.

Study participants are asked to return to the study site for 2 further visits, at day 84 and day 168 (Figure 1), and to bring their diaries and medication with them (whether used, partially used, or not used). At visit 2, researchers check the diaries to verify that participants are completing them correctly and provide them with new diaries and another 3 cycles of NGM/EE 0.25/0.035 mg. At both visits 2 and 3, the same assessments are conducted as at the baseline visit (physical examination, GAGS, POMS, FSFI, and dysmenorrhea VAS), and participants are asked about concomitant treatments, adherence, and AEs. At visit 3, the diaries are collected for final verification, and the subgroup of participants from Genoa provides another blood sample. In the case of an early withdrawal before the end of the sixth cycle, the investigator asks the participant to return for a final visit and records the reasons for the participant’s decision to discontinue the study.

**Parameters evaluated in blood samples in the subgroup of participants from Genoa.**

**Metabolic**

- Glucose
- Insulin
- Total cholesterol
- High-density lipoprotein cholesterol
- Low-density lipoprotein cholesterol
- Triglycerides

**Endocrine**

- Total testosterone
- Dehydroepiandrosterone
- Androstenedione
- Sex hormone–binding globulin
- Free androgen index

**Related to coagulation**

- Fibrinogen
- Factor VII
- Antithrombin III
- Factor VIII
- Protein C
- Thrombin time
- Anticoagulant functional protein C
- Total protein S
- Activated protein C resistance

**Table 1:** Cycle evaluation parameters.

Parameter	Normal	Abnormal
Frequency	≥24 and ≤38 days	Absent (no bleeding): amenorrhea Infrequent (>38 days)
Duration	≤8 days	Prolonged (>8 days)
Regularity	Regular (shortest to longest cycle variation: ≤7 to 9 days^a^)	Irregular (shortest to longest cycle variation: ≥10 days)
Flow volume (participant determined)	Participant considers normal	Participant considers light Participant considers heavy
Breakthrough bleeding (bleeding or spotting between the cyclically regular onset of menses)	None	Random Cyclical (predictable): Early cycle Mid cycle Late cycle
Unscheduled bleeding on progestin±estrogen gonadal steroids (contraceptive pills, rings, patches, intrauterine contraceptive devices, or injections)	None (for a participant on gonadal steroid medication) Not applicable for participants not on gonadal steroid medication	Present

^a^Normal variation depends on age; these data are calculated excluding short and long outliers.

### Data Management

Investigators collect the information in a validated electronic data system managed by a contract research organization (Advice Pharma Group Srl). The contract research organization is responsible for checking the completeness of the data, monitoring the quality of the data, identifying any extreme values (outliers), and storing the information securely according to legal requirements. Access to the data is strictly limited to authorized personnel only, and individual patient information is deidentified prior to analysis.

### End Points

The primary objective of the study is to investigate menstrual cycle control by evaluating breakthrough bleeding (ie, bleeding or spotting between the cyclically regular onset of menses) during the use of the monophasic NGM/EE 0.25/0.035 mg COC. This is measured by calculating the intermenstrual bleeding or spotting occurrence rate at the sixth cycle and comparing this with the rate at baseline.

Secondary outcomes include the change from baseline at visits 2 and 3 in other parameters of cycle control (frequency, duration, regularity, participant-determined flow volume, and unscheduled bleeding), GAGS score, POMS, FSFI, and dysmenorrhea severity based on the VAS rating. Adherence and contraceptive failure rate over the 6-month study period are also secondary outcomes, as are changes from baseline in the metabolic, hormonal, or coagulation parameters in the subgroup of participants from Genoa. Adherence is being evaluated as a percentage of tablets taken, based on the medication returned by participants at each visit. Contraception failure is defined as the proportion of women who become pregnant during the 6-month study period. The reasons for any contraception failure will be identified and listed (eg, discontinuation or poor adherence).

### Safety Outcomes

AEs, including any serious adverse events (SAEs), are the key safety outcome. An AE can be any unfavorable and unintended sign (including an abnormal laboratory finding), symptom, or disease temporally associated with the use of NGM/EE 0.25/0.035 mg, whether considered related or not. This may include exacerbation of existing conditions, suspected drug-drug interactions, or clinically significant abnormal laboratory findings, although an abnormal laboratory finding may not be considered as an AE if there is no change compared to baseline values. Abnormal laboratory values or test results represent an AE only if they induce clinical signs or symptoms, are clinically significant, or require therapy. An SAE is any AE that results in death, is life-threatening, requires inpatient hospitalization or prolongation of an existing hospital stay, results in persistent or significant disability or incapacity, or results in a congenital anomaly or birth defect. Other medical events that may jeopardize the participant or may require a medical or surgical intervention to prevent an SAE are also considered to be serious.

Investigators will record any AEs or SAEs in a case report form, with a description of the event, date of occurrence, an assessment of its severity and potential relationship to NGM/EE 0.25/0.035 mg, and the outcome (including date of resolution). If there is at least a reasonable possibility that NGM/EE 0.25/0.035 mg is the cause of the AE (ie, that causality cannot be ruled out), the AE will be considered as an adverse drug reaction. The severity of the AE will be rated as mild (transient and generally not interfering with usual activities), moderate (sufficiently discomforting to interfere with usual activities), or severe (inability to perform usual activities). AEs of special interest in this study are hypertension, arterial thromboembolism, myocardial infarction, stroke, transient ischemic attacks, venous thrombosis and pulmonary embolism, liver and breast tumors, cancer of the cervix, and disturbances in liver function (such as increased hepatic enzyme levels).

### Statistical Analysis

Based on the previous large-scale study with NGM/EE 0.25/0.035 mg, the rate of breakthrough bleeding is expected to decrease from 4.5% before use to 3% after 6 cycles, and the rate of intermenstrual spotting to decrease from 9% to 4% [17]. Therefore, a sample size of 175 participants would have 80% power to detect a treatment effect on breakthrough bleeding at a 1-sided α level of 5%. To account for a potential dropout rate of 30%, we plan to screen 240 women for eligibility and enroll 228 participants, an average of 38 participants at each of the 6 centers.

Two datasets have been defined for analysis: the per-protocol set, which includes all participants with full treatment adherence and no major protocol deviations, and the intention-to-treat set, which includes all enrolled participants who received at least 1 dose of NGM/EE 0.25/0.035 mg. The primary outcome will be evaluated only in the per-protocol population to avoid any effects of noncompliance on the interpretation of this outcome. However, secondary efficacy outcomes and safety will be assessed in the intention-to-treat population using robust statistical methods against missing data, like linear mixed models.

Continuous variables will be described using the mean and standard deviation if they are normally distributed or using the median and interquartile range if nonnormally distributed. The distribution of these variables will be assessed using the 2-tailed Student *t* test for paired data or the Wilcoxon signed rank test. Categorical variables will be described using frequencies and percentages and compared using the chi-square test or Fisher exact test [25]. There will be no imputation for missing data, and data from unscheduled visits will not be included in the analysis. No adjustment for multiplicity will be made to adjust for a type I error in secondary end points. A *P* value of .05 is considered to be statistically significant.

All statistical analyses will be performed using the R statistical software (version 3.5 or later; R Foundation for Statistical Computing). The final analysis will be completed after all participants have completed the study, any queries have been resolved, and the database has been locked.

## Results

Study enrollment began in July 2023 and is expected to be complete by December 2025. The last participant’s last visit is anticipated to be in June 2026, and data analysis will be complete by October 2026.

## Discussion

### Overview

We anticipate that our study will confirm previous research [17,18], showing that monophasic COC with NGM/EE 0.25/0.035 mg reduces breakthrough bleeding in healthy women. However, our study will also investigate other effects of this COC on menstrual characteristics (regularity, flow volume, and dysmenorrhea). Moreover, the study will assess the impact of NGM/EE 0.25/0.035 mg on the other factors women want from their oral contraceptives in terms of pregnancy prevention, safety, and tolerability [14].

The absolute risk of VTE is 6.29 per 10,000 woman-years in women taking COCs, which while being twice the rate in women not taking COCs is still low [26]. Although the risk is low, it is still present; therefore, physicians should be vigilant to the potential for this AE among their patients taking COCs. The risk of VTE appears to be lower with NGM than with many other types of progestin [12,13], so we do not anticipate the development of VTE among women participating in this study, which is probably too small and of too short a duration to estimate the incidence of VTE associated with NGM/EE 0.25/0.035 mg. Nevertheless, thromboembolism of any type is an AE of special interest in the study, and any occurrence will be thoroughly investigated and reported.

Our study includes an evaluation of the effects of NGM/EE 0.25/0.035 mg on acne using the GAGS. It is expected that acne will improve during the use of monophasic NGM/EE 0.25/0.035 mg since the exacerbation of acne is usually related to the androgenic potency of the COC [27].

### Comparison to Prior Work

The previous large-scale study conducted in Germany among 59,701 women evaluated 342,348 menstrual cycles and showed a reduction in breakthrough bleeding from 4.5% at baseline to 3% during cycle 6 of the COC and in intermenstrual spotting from 9% to 4% during cycle 6 [16]. In the Italian study by Affinito et al [18], in which 92 women received monophasic NGM/EE, the incidence of breakthrough bleeding decreased from 3.3% to 0% at cycle 6, and the rate of spotting from 14.3% to 3.7%. Our study will include at least twice the number of women as in the earlier Italian study and therefore a more robust assessment of the effects of NGM/EE on these menstrual characteristics.

The large German study highlighted the extremely high contraceptive efficacy of NGM/EE, showing a use-efficacy Pearl index of 0.25 (95% CI 0.19-0.31) pregnancies per 100 woman-years [17], and no pregnancies were reported in the earlier Italian study [18]. There was a low incidence of AEs and a high rate of compliance in both these analyses [17,18]. Our study will provide updated information on the contraceptive efficacy, adherence rate, and safety or tolerability profile of NGM/EE 0.25/0.035 mg. The substudy is investigating the effect of NGM/EE 0.25/0.035 mg on lipid and metabolic parameters, expected to be negligible based on previous research [17,18].

### Strengths and Limitations

A key difference between our study and the earlier ones is the incorporation of PROs to gather subjective information about the impact of monophasic NGM/EE on the women who take it, specifically the impact on sexuality, mood, and menstrual pain. The inclusion of PROs in our study predated, but is nevertheless consistent with, international consensus recommendations on research into contraceptive-induced menstrual changes, which recommend investigating the physical and psychosocial impact of these changes on the lives of the women who take them, including their sexual well-being and anxiety or stress [19].

Sexual side effects are a leading reason for women to discontinue or switch contraceptives [28], so it is important to ask women about the effect of COCs on their sexual function. The reported impact of COCs on sexual function is highly variable and encompasses negative, positive, and no effects [29,30]. Female sexuality is complex, and the association between hormone levels and sexuality is nonlinear and multidimensional since a number of different hormones regulate sexual response [29]. Data suggest that the androgenicity of the progestin and the dosage of EE in COCs have a negligible impact on sexual function [31]. Based on the available literature, we do not anticipate a significant effect of NGM/EE 0.25/0.035 mg on sexual function, but to our knowledge, this is one of the first studies to investigate the effects of this COC on sexuality using a validated instrument, the FSFI.

We are also investigating any changes in mood after 6 cycles of NGM/EE 0.25/0.035 mg using the POMS. This instrument has been used previously to investigate changes in mood during oral contraceptive use [32,33], but to our knowledge, this is the first study to use POMS to evaluate mood in women taking NGM-containing COCs.

Finally, our study includes an assessment of the severity of pain associated with menstruation in accordance with international consensus recommendations [19]. Previous studies have evaluated the incidence of pain in women taking monophasic NGM/EE but not the severity of pain [17,18]. This is an important distinction because pain is a continuum rather than a binary outcome. Data indicate that at least 90% of women experience some kind of discomfort during menstruation [34], although their experiences vary across time [35]. Dysmenorrhea is often underreported for many reasons, including that symptoms are considered normal or that women find the symptoms tolerable [36]. Therefore, pain was likely underreported in clinical trials because participants may only report pain that is new, worse, or acute [34]. Using a tool like VAS will provide nuanced information about both the incidence and severity of menstrual pain in women taking NGM/EE 0.25/0.035 mg. Limitations of our study are the absence of a control group and the relatively short duration of follow-up.

### Conclusions

This study into the effects of monophasic NGM/EE 0.25/0.035 mg on menstrual characteristics in healthy Italian women will provide up-to-date data on these effects, since there has been no similar study for more than 30 years, and includes the assessment of NGM/EE 0.25/0.035 mg on PROs, in line with recent international consensus recommendations.

## Abbreviations

AE: adverse event
COC: combined oral contraceptive
EE: ethinyl estradiol
FSFI: Female Sexual Function Index
GAGS: Global Acne Grading Scale
NGM: norgestimate
POMS: Profile of Mood State
PRO: patient-reported outcome
SAE: serious adverse event
VAS: visual analog scale
VTE: venous thromboembolism
